# The Role of Community Cohesion in Older Adults During the COVID-19 Epidemic: Cross-sectional Study

**DOI:** 10.2196/45110

**Published:** 2023-04-14

**Authors:** Ying Li, XiWen Ding, Ayizuhere Aierken, YiYang Pan, Yuan Chen, DongBin Hu

**Affiliations:** 1 Department of Social Medicine, School of Public Health Zhejiang University HangZhou China; 2 School of Medicine, Zhejiang University HangZhou China; 3 Department of Quality Surveillance of Zhejiang Provincial People's Hospital HangZhou China; 4 The Second Affiliated Hospital, Harbin Medical University Harbin China

**Keywords:** community cohesion, physical and mental health, community services, environmental resources, COVID-19 epidemic, older adults

## Abstract

**Background:**

The community environment plays a vital role in the health of older adults. During the COVID-19 epidemic, older adults, who were considered the most impacted and most vulnerable social group, were confined to their homes during the implementation of management and control measures for the epidemic. In such situations, older adults may have to contend with a lack of resources and experience anxiety. Therefore, identifying the environmental factors that are beneficial for their physical and mental health is critical.

**Objective:**

This study aimed to assess the association between community cohesion and the physical and mental health of older adults and to identify the related community services and environmental factors that may promote community cohesion.

**Methods:**

This community-based cross-sectional study was designed during the COVID-19 epidemic. A multistage sampling method was applied to this study. A total of 2036 participants aged ≥60 years were sampled from 27 locations in China. Data were collected through face-to-face interviews. The neighborhood cohesion instrument consisting of scales on 3 dimensions was used to assess community cohesion. Self-efficacy and life satisfaction, cognitive function and depression, and community services and environmental factors were also measured using standard instruments. Statistical analyses were restricted to 99.07% (2017/2036) of the participants. Separate logistic regression analysis was conducted to assess the association among community cohesion and physical and mental health factors, related community services, and environmental factors among older adults.

**Results:**

The results showed that high levels of community cohesion were associated with good self-perceived health status and life satisfaction (odds ratio [OR] 1.27, 95% CI 1.01-1.59 and OR 1.20, 95% CI 1.15-1.27, respectively) and high levels of self-efficacy and psychological resilience (OR 1.09, 95% CI 1.05-1.13 and OR 1.05, 95% CI 1.03-1.06, respectively). The length of stay in the community and the level of physical activity were positively associated with community cohesion scores, whereas the education level was negatively associated with community cohesion scores (*P*=.009). Community cohesion was also associated with low levels of depression and high levels of cognitive function. Community cohesion was significantly associated with community services and environmental factors on 4 dimensions. High levels of community cohesion were associated with transportation services and rehabilitation equipment rental services as well as high levels of satisfaction with community physicians’ technical expertise and community waste disposal (OR 3.14, 95% CI 1.87-5.28; OR 3.62, 95% CI 2.38-5.52; OR 1.37, 95% CI 1.08-1.73; and OR 1.23, 95% CI 1.01-1.50, respectively).

**Conclusions:**

Community cohesion was found to be associated with the physical and mental health of older adults. Our research suggests that enhancing community services and environmental resources may be an effective strategy to increase community cohesion during major infectious disease epidemics.

## Introduction

### Background

Older adults become more vulnerable to mental diseases and environmental challenges as their age increases [1]. The community environment plays a vital role in their health, especially their mental health, as they age [2]. They usually wish to remain living in the community as long as possible, but their health status may deteriorate during the last stage of their lives to the point where they can no longer live alone. Therefore, the World Health Organization suggests the provision of a familiar environment and an emotional bond to support *aging in place* to enable older adults to live in their community independently and comfortably by experiencing a sense of attachment, connectedness, security, and identity [3-5].

During the COVID-19 pandemic, older adults were considered the most impacted and vulnerable social group because they had a high risk of being infected by SARS-CoV-2 and dying [6]. Several epidemic prevention and control measures had changed to a large extent the way of living they were accustomed to and seriously interfered with their access to public resources. During the initial stage of the pandemic, they were alone and unattended at home; thus, they could not purchase masks and obtain any information on disease prevalence; they also felt anxious, helpless, and scared [7-9]. Some studies showed that the deterioration in physical functioning and the reduction in social contact may have caused many older adults to experience poor mental health [10,11]. During COVID-19–related lockdowns imposed by the authorities, older adults were cut off from the outside world, which also hindered them from receiving social support [12]. In such circumstances, older adults can experience serious physical and mental health consequences, and, as a result, they become more dependent on community services and environment resources.

### The Importance of Community Cohesion

Community cohesion, which is an important element of the community, includes 3 essential dimensions. It involves social relations, identification with the geographical unit, and orientation toward the common good [13]. Individual-level community cohesion is assessed in a geographically bounded neighborhood; this is known as the community or neighborhood cohesion assessment. In recent years, interest in the study of community cohesion has escalated, probably because of the sharp increase in older populations. A study suggested that community cohesion is an important factor that affects physical and psychological health outcomes among older adults [14]. Many studies have shown that community cohesion may positively influence both friendship and well-being, which act as buffers against serious psychological distress among older adults [15-17]. A lack of community cohesion is associated with depression, loneliness, suicidal ideation, and poor mental health [18-20]. Community cohesion is also an important protective factor for preventing posttraumatic stress disorder symptoms when older adults experience negative events and disasters [21]. Clinical and epidemiological studies have found a lack of community cohesion to be associated with disability, hypertension, myocardial infarction, stroke, and mortality [22-25]. Empirical studies have confirmed that low levels of community cohesion can accelerate epigenetic aging, and high levels of community cohesion can reduce the risks of diseases associated with genetic risk factors [26,27].

The COVID-19 pandemic has had an enormous and long-lasting negative impact on the physical and mental health of older adults worldwide. However, the unprecedented COVID-19 outbreak and special prevention and control measures have resulted in a limited number of studies being conducted on the association of community cohesion with the physical and mental health of older adults. We found several similar studies on the relationship between community cohesion and mental health, but these studies have a small sample size, resulting in low statistical power [28,29]. In addition, the difficulty in data collection during the COVID-19 pandemic resulted in only a single study (with a lack of theoretical models) being conducted on the association of community cohesion with the mental health of older adults. A multiscale evaluation study on the complex impact of the COVID-19 pandemic on the physical and mental health of older adults has not yet been conducted. To our knowledge, no research has assessed community cohesion factors, such as community services and community resources, that affected the physical and mental health of older adults and the possible community public strategies that could have been used to improve their physical and mental health during the COVID-19 pandemic while at the emergency prevention and control stage. Through this research, we expected to collect considerable amounts of community resources information as well as physical and mental health measurement data so that we could fully explore the effects of community cohesion on the physical and mental health of older adults at the individual, community, and social levels during the COVID-19 pandemic based on the social-ecological model and investigate the complex relationship among community cohesion, community services, environmental resources, and the physical and mental health of older adults and the potential interaction mechanism.

We hypothesized that a good community environment and satisfactory health service facilities can improve community cohesion and enhance community residents’ perception of social support and self-efficacy in the effective use of community services and environmental resources. This study aimed to explore the effects of community cohesion on the physical and mental health of older adults living in a community during the COVID-19 epidemic, identify the association between community cohesion and perceived social support and self-efficacy, and clarify and define the community services and environmental factors that may promote community cohesion.

## Methods

### Study Design

A cross-sectional study was conducted among community-dwelling older adults to assess the related factors of community cohesion and the association between community cohesion and the physical and mental health of older adults during the COVID-19 pandemic. This study was supported by a big data–driven community mental health management model as well as an accessibility evaluation of health-related resource projects. Two community-based surveys that aimed to assess health status and accessibility of community health services among older adults living in the community were conducted to discover the main psychological and mental health challenges and explore ways to promote community health based on the theory of social ecology.

### Participants

A total of 2036 community residents were selected through a multistage sampling design according to comprehensive geographic location attributes and fully considering the regional aging degree. Sampling was performed at 27 locations in 4 provinces in China from July 15, 2020, to August 31, 2022. All participants were aged ≥60 years. Participants with severe physical dysfunction and mental disorders who would not have been able to complete the questionnaire were not eligible to participate in this study.

### Ethics Approval and Participation

The study was approved by the institutional review board at the School of Public Health, Zhejiang University (ZGL2020-010), and it was performed in accordance with the principles of the Declaration of Helsinki. All participants provided written informed consent; the secondary analysis was allowed without additional consent. All data were anonymous and kept confidential to protect the privacy of participants. This study was required to pay ≥CN¥40 (US $5.8) to each participant.

### Data Collection

Well-trained investigators conducted face-to-face interviews with all participants in the presence of family members or in the community. Before the formal survey, community administrators made a home visit or telephone call to older adults living in the community. The community administrators briefly explained the basic situation and recruitment requirements for the project to the older adults and asked them whether they would participate in the survey. Participants who agreed were then scheduled to take the survey. On the day of the survey, the investigator explained the purpose, content, method, and other information regarding the survey to the eligible participants. Subsequently, the investigator received the participants’ informed consent. Most of the participants (1835/2036, 90.13%) completed the interview in 50-60 minutes. The questionnaires consisted of 16 parts and included 578 items. The main contents included the following information: demographic characteristics; behavioral habits and general health status; community services and environmental resources; community cohesion; social support and psychological resilience; self-efficacy; neurocognition and social cognition; and assessment of depression, personality disorder, and activities of daily living.

The following general characteristics of the participants were collected: age, sex, education level, individual monthly income, marital status, physical activity level, and dietary habit. Community services and environmental resources were assessed in terms of 4 dimensions: community service facilities, medical resources, nursing resources, and welfare resources. Smoking status was examined using the question *Do you smoke?* If the response was yes, the question *What kind of cigarette do you smoke?* was asked, followed by the question *How many cigarettes do you smoke in a day?* Smoking history and age of starting smoking were also included in the questions. Physical activity status was examined using the question *Do you regularly participate in sports, such as hiking, jogging, playing ball, or swimming?* If the response was yes, details regarding time spent and frequency of participation in physical activity were obtained. Sleep was assessed using the following questions: *How many hours of actual sleep did you have per night during the past month?* and *When do you usually go to bed and get up?* Regarding daily living habits, participants were asked the following questions: *Do you eat at regular times every day? Do you have the habit of drinking tea?* and *Do you have a mobile phone?* Self-perceived health status was assessed using the question *Would you say your health is very good, good, fair, poor, or very poor?* Individuals with chronic diseases were identified using the question *Have you ever been diagnosed with chronic diseases by a doctor?* A total of 28 chronic diseases were included in the options.

Public service facilities in the community were assessed by using the questions *Does your community provide a meal service? Are activity centers for older adults available near your community?* and *Are you satisfied with the clearing and disposal of garbage?* The answer options were *Yes* and *No*. Community medical service resources were assessed using the questions *How far is your home from the nearest medical institution?* and *Does your community provide a family doctor contract service?* Nursing resources were assessed using the questions *Does your community provide day care services?* and *Does your community provide nursing care for patients with severe chronic disease?* The questions used to determine welfare resources included *Does your community organize regular outdoor activities for older adults?* and *Does your community provide transportation services?*

### Measurements

#### Community Cohesion

The neighborhood cohesion instrument was applied to assess community cohesion using 18 items [30]. The neighborhood cohesion instrument consists of scales on 3 dimensions to measure the synthesis of community cohesion concepts. The 3 items—*Overall, I am very attracted to living in this neighborhood*; *Given the opportunity, I would like to move out of this neighborhood*; and *I rarely have neighbors come over to my house to visit*—measure attraction to the neighborhood. Six items, including *If I needed advice about something I could go to someone in my neighborhood*, *I borrow things and exchange favors with my neighbors*, and *I rarely have neighbors come over to my house to visit*, measure the degree of neighboring. Nine items, including *I feel like I belong to this neighborhood*, *I think I agree with most people in my neighborhood about what is important in life*, and *I feel loyal to the people in my neighborhood*, measure the psychological sense of community. The response options for each item are (1) strongly agree, (2) agree, (3) neither agree nor disagree, (4) disagree, and (5) strongly disagree based on a 5-point Likert scale. The estimated Cronbach α coefficients range from .86 to .95.

#### Other Measurements

The general self-efficacy scale was used to measure the level of self-efficacy [31]. The general self-efficacy scale consists of 10 items with a total score ranging from 10 to 40 points; the higher the score, the higher the level of self-efficacy. The Chinese version of the Older American Resources and Services (OARS) scale was used to assess the level of social support [32]. The OARS scale consists of 3 dimensions: social interaction, family support, and interpersonal relations. High OARS scores indicate high levels of social support. The Chinese version of the 21-item Dementia Assessment Sheet for Community-Based Integrated Care System (DASC-21) was used to assess individual cognitive functions [33]. A score of ≥27 points suggests possible dementia, and a high score is recognized as indicative of a low level of cognitive function. The Eysenck Personality Questionnaire-Revised Short Scale was used to assess the personality characteristics of participants [34]. Community services and environmental resources included 44 items. However, the item *Help with using the toilet* was removed, given that participants with severe physical dysfunction were not eligible to participate in the study [35].

### Statistical Analysis

The data obtained from participants with complete questionnaires (N=2017) were used for statistical analysis. Descriptive statistics were used to report the general characteristics of participants. Frequencies and percentages were computed for the variables.

The means and SDs of community cohesion scores were calculated using participant characteristics. A 2-tailed *t* test was used to compare the mean values between 2 groups, and 1-factor ANOVA was used to compare multiple groups. We conducted a normality test and a variance homogeneity test before performing the *t* test and 1-factor ANOVA.

Three separate logistic regression analyses were conducted to evaluate the association between community cohesion and physical and mental health factors by area (urban vs rural). Community cohesion scores were treated as a binary dependent variable and added to the logistic regression model. Self-perceived health and life satisfaction, cognitive function and depression, and self-efficacy and psychological resilience were added as independent variables to the 3 logistic regression models. Age, sex, marital status, education level, individual monthly income, physical activity level, and dietary habit were adjusted in all models, and the Eysenck Personality Questionnaire-Revised Short Scale scores for psychological factors were also controlled.

Binary logistic regression analysis was used to assess the association between community cohesion and self-perceived social support. The scores of the 3 dimensions for social support and the total scores were added to the logistic regression model. The association of community cohesion with community environmental factors was assessed on 3 dimensions—attraction to neighborhood, degree of neighboring, and psychological sense of community—using binary logistic regression analysis. The total community cohesion score, which was divided into 2 categories, was used as a dependent variable to establish the logistic regression model. For each logistic regression model, we used the stepwise regression method to select the variables. A variable has a power of 80% at a significance level of .05 for univariate analyses that can be added to the logistic regression model. The community cohesion scores among different levels of community services and environmental resources were calculated, and a radar map was drawn.

The association among community cohesion and self-efficacy, social support, cognitive function, community services and environmental resources, and physical and mental health was evaluated through mediating and moderating effect tests based on step-by-step general linear regression model. Statistical significance was set at *P*<.05 for the 2-tailed test. All data analyses were performed using SAS for Windows (version 9.4; SAS Institute Inc).

## Results

### Participant Characteristics

The general characteristics of participants by sex are shown in Table 1. Of the 2017 participants, 855 (42.39%) were aged ≥70 years, 771 (38.22%) were male, and 1246 (61.77%) were female. A total of 20.48% (413/2017) of the participants reported that they had completed ≥13 years of education. In terms of marital status, 9.5% (73/771) of the male participants and 23.43% (292/1246) of the female participants were unmarried. Overall, 72.93% (1471/2017) of the participants self-reported that they had ≥1 chronic diseases. More than half of the participants (1108/2017, 54.93%) had lived in their community for ≥30 years.

**Table 1:** Characteristics of study participants by sex (N=2017).

Variable categories		Male participants (n=771), n (%)					Female participants (n=1246), n (%)
**Age (years)**							
	<70	399 (51.8)					763 (61.23)
	≥70	372 (48.2)					483 (38.76)
**Years of education**							
	0-6		72 (9.3)				231 (18.53)
	7-9		314 (40.7)				464 (37.23)
	10-12		216 (28.1)				307 (24.63)
	≥13		169 (21.9)				244 (19.58)
**Individual monthly income (CN¥ [US $])**							
	0-1999 (0-291)					256 (33.2)	362 (29.05)
	2000-3999 (292-584)					281 (36.5)	621 (49.83)
	≥4000 (585)					234 (30.3)	186 (14.92)
**Marital status**							
	Married			698 (90.5)			954 (76.56)
	Unmarried			73 (9.5)			292 (23.43)
**Smoking status**							
	Smoker				364 (47.2)		19 (1.52)
	Nonsmoker				407 (52.8)		1227 (98.48)
**Alcohol use**							
	Yes					419 (54.4)	152 (12.19)
	No					234 (45.6)	1094 (87.80)
**Physical activity**							
	Yes				390 (50.6)		794 (63.72)
	No				381 (49.4)		452 (36.28)
**Chronic disease status**							
	Yes		579 (75.1)				892 (71.59)
	No		192 (24.9)				354 (28.41)
**Years lived in the community**							
	<30			299 (38.8)			610 (48.96)
	≥30			472 (61.2)			636 (51.04)

### Community Cohesion Scores Based on Participant Characteristics

Table 2 shows the means and SDs of community cohesion scores based on the characteristics of the participants. According to the logistic regression analysis, education level was negatively associated with the community cohesion score: the lower the education level, the higher the community cohesion score. In addition, we used a generalized linear regression model, and we observed a strong statistically significant linear trend (*P*<.001). Participants’ self-reported levels of physical activity were positively associated with community cohesion scores (*P*=.003). The community cohesion scores were higher (*P*<.001) when individuals lived in the community longer.

**Table 2:** The means and SDs of community cohesion scores by participant characteristics.

Variable categories		Values, mean (SD)		*P* value
**Age (years)**				.39
	<70	68.4 (9.5)		
	≥70	69.1 (8.3)		
**Sex**				.01
	Male	67.3 (8.2)		
	Female	69.3 (9.2)		
**Years of education**				<.001
	0-6	71.6 (9.3)		
	7-9	70.5 (8.2)		
	10-12	68.1 (9.6)		
	≥13	65.6 (7.9)		
**Individual monthly income (CN¥ [US $])**				<.001
	0-1999 (0-291)	65.3 (8.8)		
	2000-3999 (292-584)	70.4 (8.7)		
	≥4000 (585)	66.9 (8.8)		
**Marital status**				.43
	Married	68.9 (9.0)		
	Unmarried	68.1 (8.5)		
**Smoking status**				.31
	Smoker	69.7 (8.6)		
	Nonsmoker	68.6 (9.0)		
**Alcohol use**				.07
	Yes		67.6 (8.7)	
	No		69.2 (9.0)	
**Physical activity**				.003
	Yes		69.3 (9.1)	
	No		66.5 (8.1)	
**Chronic disease status**				.41
	Yes		68.6 (8.8)	
	No		69.3 (9.3)	
**Years lived in the community**				<.001
	<30		67.2 (8.9)	
	≥30		70.8 (8.6)	

### Community Cohesion–Related Physical and Mental Health Status

The evaluation of community cohesion–related physical and mental health status by area (urban vs rural) is presented in Table 3. The logistic regression analysis showed that high levels of community cohesion were positively associated with life satisfaction, psychological resilience, levels of self-efficacy, and self-perceived health status (odds ratio [OR] 1.21, 95% CI 1.14-1.28; OR 1.05, 95% CI 1.03-1.07; OR 1.11, 95% CI 1.06-1.16; and OR 1.36, 95% CI 1.04-1.78, respectively, in urban areas; and OR 1.15, 95% CI 1.03-1.28; OR 1.07, 95% CI 1.02-1.11; OR 1.02, 95% CI 1.01-1.07; and OR 1.14, 95% CI 0.90-1.83, respectively, in rural areas). High levels of community cohesion were associated with good levels of cognitive function and low levels of depression, and community cohesion was negatively associated with cognitive function and depression scores (OR 0.92, 95% CI 0.87-0.98 and OR 0.80, 95% CI 0.71-0.90, respectively, in urban areas; and OR 0.88, 95% CI 0.79-0.91 and OR 0.92, 95% CI 0.90-0.99, respectively, in rural areas).

**Table 3:** Community cohesion–related physical and mental health factors by logistic regression analysis.

Variables		Multivariable adjusted odds ratio (95% CI)	*P* value
**Urban areas**			
	Life satisfaction	1.21 (1.14-1.28)	<.001
	Cognitive function	0.92 (0.87-0.98)	<.001
	Depression	0.80 (0.71-0.90)	<.001
	Self-efficacy	1.11 (1.06-1.16)	.002
	Psychological resilience	1.05 (1.03-1.07)	<.001
	Self-perceived health	1.36 (1.04-1.78)	.03
**Rural areas**			
	Life satisfaction	1.15 (1.03-1.28)	<.001
	Cognitive function	0.88 (0.79-0.91)	.02
	Depression	0.92 (0.90-0.99)	.04
	Self-efficacy	1.02 (1.01-1.07)	.05
	Psychological resilience	1.07 (1.02-1.11)	.02
	Self-perceived health	1.14 (0.90-1.83)	.06

### Association Between Community Cohesion and Self-perceived Social Support

Table 4 shows a positive association between community cohesion and self-perceived social support after education level, dietary habit, age, sex, marital status, physical activity level, and individual monthly income were adjusted (OR 1.27, 95% CI 1.15-1.40; *P*<.001). Community cohesion was significantly associated with the 3 dimensions of social support: social interaction, family support, and interpersonal relations (OR 1.39, 95% CI 1.18-1.65; OR 1.34, 95% CI 1.05-1.72; and OR 1.31, 95% CI 1.13-1.52, respectively).

**Table 4:** The association between community cohesion and self-perceived social support.

Variables	Multivariable adjusted odds ratio (95% CI)	*P* value
Total scores of social support	1.27 (1.15-1.40)	<.001
Social interaction	1.39 (1.18-1.65)	<.001
Family support	1.34 (1.05-1.72)	.02
Interpersonal relations	1.31 (1.13-1.52)	<.001
Education level	0.72 (0.56-0.92)	.009
Dietary habit	0.85 (0.57-1.27)	.41
Sex	1.15 (0.70-1.89)	.59
Age	1.02 (0.98-1.06)	.32
Marital status	0.63 (0.33-1.19)	.16
Physical activity	1.06 (0.63-1.80)	.83
Individual monthly income	0.84 (0.63-1.11)	.21

### Association Between Community Cohesion and Community Services and Environmental Factors

The community cohesion scores from different dimensions associated with community services and environmental factors by logistic regression analysis are shown in Table 5. In model 1, attraction to neighborhood was significantly associated with levels of satisfaction with community physicians’ attitude, rehabilitation equipment rental services, caregiver guidance, and community waste disposal (OR 1.55, 95% CI 1.18-2.05; OR 3.91, 95% CI 2.39-6.37; OR 1.85, 95% CI 1.08-3.17; and OR 1.37, 95% CI 1.10-1.71, respectively). In model 2, the degree of neighboring was associated with re-employment assistance, rehabilitation equipment rental services, and levels of satisfaction with community physicians’ technical expertise (OR 2.49, 95% CI 1.49-4.18; OR 1.63, 95% CI 1.10-2.42; and OR 1.24, 95% CI 1.01-1.53, respectively). In model 3, psychological sense of community was associated with transportation services, rehabilitation equipment rental services, and health assessment (OR 2.96, 95% CI 1.78-4.92; OR 3.55, 95% CI 2.34-5.39; and OR 2.27, 95% CI 1.28-4.04, respectively). In model 4, the overall levels of community cohesion were associated with transportation services and rehabilitation equipment rental services as well as levels of satisfaction with community physicians’ technical expertise and community waste disposal (OR 3.14, 95% CI 1.87-5.28; OR 3.62, 95% CI 2.38-5.52; OR 1.37, 95% CI 1.08-1.73; and OR 1.23, 95% CI 1.01-1.50, respectively).

**Figure 1:**
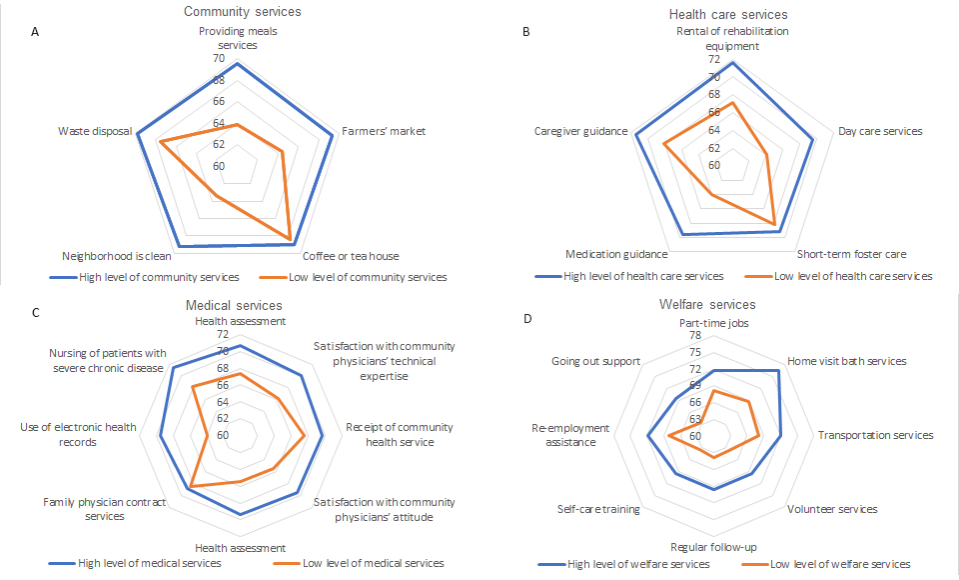
Mean community cohesion scores. (A) Community cohesion scores for public services. (B) Community cohesion scores for health care services. (C) Community cohesion scores for medical services. (D) Community cohesion scores for welfare services.

The mean scores of community cohesion from the 4 dimensions of community services and environmental resources are shown in Figure 1. In the community services environment, provision of meals, use of farmers’ markets, and neighborhood showed high community cohesion scores (Figure 1A). In health care services, high community cohesion scores were observed in the following services: day care service, medication guidance, rehabilitation equipment rental services, and caregiver guidance (Figure 1B). In medical services, health assessment, satisfaction with community physicians’ attitude, receipt of community health service, satisfaction with community physicians’ technical expertise, nursing of patients with severe chronic disease, and use of electronic health records showed high community cohesion scores (Figure 1C). In welfare services, home visit bath service, transportation service, volunteer service, regular follow-ups, self-care training, re-employment assistance, going out support, and part-time jobs were associated with high community cohesion scores (Figure 1D).

**Table 5:** The odds ratios of community environment and other factors related to community cohesion by logistic regression model.

Variables		Multivariable adjusted odds ratio (95% CI)			*P* value		
**Model 1 (attraction to neighborhood)**							
	Levels of satisfaction with community physicians’ attitude			1.55 (1.18-2.05)			.001
	Levels of satisfaction with community waste disposal			1.37 (1.10-1.71)			.005
	Rehabilitation equipment rental services			3.91 (2.39-6.37)			<.001
	Caregiver guidance			1.85 (1.08-3.17)			.03
	Depression scores			0.85 (0.76-0.94)			.001
	EPQ-RSS^a^ scores			1.09 (1.03-1.16)			.004
**Model 2 (degree of neighboring)**							
	Levels of satisfaction with community physicians’ technical expertise		1.24 (1.01-1.53)			.049	
	Rehabilitation equipment rental services	1.63 (1.10-2.42)			.02		
	Re-employment assistance	2.49 (1.49-4.18)			<.001		
	Depression scores	0.90 (0.82-0.99)			.04		
	Age	0.96 (0.94-0.98)			.006		
**Model 3 (psychological sense of community)**							
	Rehabilitation equipment rental services		3.55 (2.34-5.39)			.005	
	Transportation services	2.96 (1.78-4.92)			<.001		
	Health assessment	2.27 (1.28-4.04)			<.001		
	Depression scores	0.86 (0.77-0.96)			.005		
	EPQ-RSS scores	1.07 (1.01-1.13)			.03		
**Model 4 (total model)**							
	Levels of satisfaction with community physicians’ technical expertise	1.37 (1.08-1.73)			.008		
	Levels of satisfaction with community waste disposal	1.23 (1.01-1.50)			.048		
	Rehabilitation equipment rental services	3.62 (2.38-5.52)			<.001		
	Transportation services	3.14 (1.87-5.28)			<.001		
	Depression scores	0.84 (0.75-0.93)			.001		
	EPQ-RSS scores	1.09 (1.04-1.16)			.001		
	Self-efficacy scores	1.05 (1.02-1.08)			.004		

^a^EPQ-RSS: Eysenck Personality Questionnaire-Revised Short Scale.

**Figure 2:**
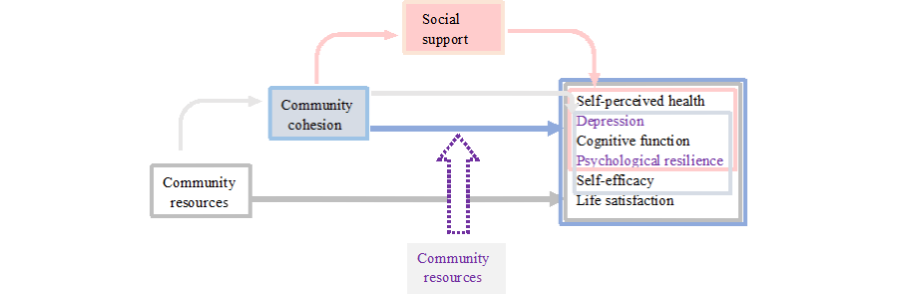
The association among community cohesion and social support, community resources, and physical and mental health of older adults.

The results of the association among community cohesion and self-efficacy, social support, cognitive function, community services and environmental resources, and physical and mental health are shown in Figure 2. Community cohesion, community resources, and social support were directly associated with physical and mental health. Community cohesion was associated with physical and mental health (self-perceived health, depression, cognitive function, and psychological resilience), which may be partially mediated by social support. Community resources were associated with physical and mental health (depression, cognitive function, psychological resilience, and self-efficacy), which may be partially mediated by community cohesion. In addition, the association between community cohesion and psychological resilience and depression may be moderated by community resources.

## Discussion

### Principal Findings

In this study, we found that community cohesion had an important effect on the physical and mental health of older adults during the COVID-19 pandemic. Moreover, high levels of community cohesion were associated with good self-perceived health status and high life satisfaction. Participants who reported high levels of community cohesion stated that they had high levels of self-efficacy, psychological resilience, and self-perceived social support. Community cohesion was positively associated with transportation and rehabilitation equipment rental services as well as levels of satisfaction with community physicians’ technical expertise and community waste disposal.

Some studies have reported the beneficial effects of community cohesion on the mental health of older adults; however, studies on the association between community cohesion and physical and mental health during the COVID-19 pandemic, especially studies on community services and environment factors that can improve community cohesion during major infectious disease epidemics, are limited [36,37]. The COVID-19 pandemic has brought about major changes in the daily lives and environment of older adults. Social support from children and relatives was unavailable because of COVID-19–related restrictions, regular visits to medical facilities were banned, and public transportation was inaccessible because they could not operate intelligent public transportation apps on their mobile phones. As they could not acquire and show health codes, as a result, they will be refused public transportation, which would lead them to feel more panicked and helpless. The restrictions on the use of public resources also caused them to become more dependent on the community. However, the implementation of COVID-19 prevention and control measures increased the interaction between the older adults and community staff, and this proved to be of considerable help to the older adults. Free testing to assess the prevalence of COVID-19 in the community made them feel safer. The community became a big presence in their lives. These pieces of evidence from the field supported our findings.

Studies have shown that rural areas have more neighborhood networks than urban areas [38], but community cohesion for health benefits between urban and rural areas demonstrates no difference among the general population. Our study showed that, during the COVID-19 pandemic, there were no differences between individuals with high levels of community cohesion association and good levels of physical and mental health in different areas (urban vs rural). However, the association between community cohesion and self-perceived health was inconsequential in rural areas. The prevalence of COVID-19 has resulted in the return of people to their community, which supports the observation on the effects of community cohesion on the physical and mental health of older adults during the COVID-19 pandemic. Our results showed that high levels of community cohesion were associated with high levels of self-perceived health status and life satisfaction. However, older adults were highly anxious about their health, and their lives were grossly circumscribed. During the pandemic, because family and social support for older adults was hindered, they became emotionally dependent on their community, and the rising levels of community cohesion enabled them to experience a high level of social support. Several studies have suggested that community cohesion can be viewed as a pattern of social support, which might affect individual mental health through enhancing mutual trust and emotional support as well as reducing stress levels [39].

Community cohesion can be linked to individual health benefits, but the underlying mechanism is still not fully understood. Studies have suggested that community cohesion enhances subjective well-being by promoting positive emotions and purpose in life, which acts as a barrier to psychopathology factors, resulting in better physical health and longevity [40-42]. Community cohesion can strengthen collective advocacy for resources, which promotes the dissemination of health-related information and increases awareness of chronic disease [43]. However, there is limited quantitative research on the association between community cohesion and physical and mental health among older adults during the COVID-19 pandemic. Our results showed that high levels of community cohesion were associated with low levels of depression and high levels of psychological resilience and cognitive function. This result can be easily explained by qualitative observation. During the pandemic, community prevention and control measures resulted in increased communication between individuals and community staff. There was also an increase in the exchange of information among neighbors on disease prevalence and prevention. In these circumstances, community cohesion and individual sense of security were enhanced, promoting the spread of health-related information and improving the recognition for diseases among older adults. A study has reported on the association between community cohesion and the use of preventive health services [44]. The study suggested that high levels of community cohesion could be positively associated with an increase in the use of influenza vaccine and cholesterol tests for community individuals through 4 hypothesized mechanisms: increasing the diffusion of information, providing emotional support, advocating for environmental resources, and maintaining healthy behaviors through informal social control.

There is growing interest in studying the effects of the environment on health and health-related outcomes, such as identifying the environmental resources and community services that may enhance community cohesion. In this study, we found that transportation and rehabilitation equipment rental services as well as levels of satisfaction with community physicians’ technical expertise and community waste disposal were positively associated with community cohesion. These factors are extremely important for improving community cohesion. During the pandemic, operating intelligent public transportation apps on their mobile phones was a huge barrier for older adults when taking public transport. Being confined to the home for long periods and the restrictions on going out led to difficulties in physical functioning, which increased the use of, and demand for, transportation and rehabilitation equipment rental services among older adults. The COVID-19 outbreak made older adults more dependent on community physicians. A high level of technical expertise demonstrated by community physicians can make older adults living in the community feel safe and reduce their fear of disease. At the initial stage of the pandemic, normal garbage disposal was also disrupted. The normalization of community garbage disposal and the clean and tidy community environment once again made the community attractive to older adults. These factors directly or indirectly affected the physical and mental health of older adults during the COVID-19 pandemic.

The main strength of this study is that it was based on the use of big data platforms to study the mental health of older adults during the COVID-19 pandemic. We collected complete data, which enabled us to consider more indicators of mental health measurement and community cohesion–related factors for the analysis and control of many potential confounding factors. The large amount of data also ensured the reliability of the study results. This study included ≥10 mental health measurement instruments and identified the association between community cohesion and the physical and mental health of older adults at the individual, community, and social levels based on the social-ecological model. Using moderation and mediation analysis, this study further revealed community cohesion to be a potential interaction mechanism linking social support, self-efficacy, environmental resources, and the physical and mental health of older adults. This study also identified specific community services and environmental factors that can increase community cohesion and that can be considered when designing public health policies to deal with major infectious disease pandemics. Furthermore, when the COVID-19 pandemic initially broke out in China, the country’s public health administration and disease control departments implemented a series of unprecedented prevention and control measures, including a *dynamic clearing* epidemic prevention policy and home isolation. This provided us with a rare opportunity to study the effects of community cohesion on the physical and mental health of older adults.

### Study Limitations

Our study includes several limitations. First, the causality could not be determined, given that this study adopted a cross-sectional design. However, the associations between community cohesion and community services and environmental factors were observed through relatively large sample sizes, and the inference was based on some previous studies. Second, all participants in this study were older adults. During the first stage of the study, the face-to-face interviews took approximately 45 minutes to complete. Each participant was compensated with CN¥40 (US $5.8), based on local research payment standards and as required by the ethics committee. After the items to be investigated were added, the survey time was extended to approximately 60 minutes, and participants were offered higher remuneration accordingly. It is possible that the compensation offered could have led prospective participants to deceive the researchers regarding their eligibility, and the information they provided could have been biased [45]. Third and last, the study was conducted during the COVID-19 pandemic. We selected the participants according to the inclusion and exclusion criteria; however, we excluded some older adults with severe physical dysfunction and mental disorders, which could have resulted in potential exclusion bias.

### Conclusions

We obtained important evidence on the effects of community services and environmental factors on community cohesion during the COVID-19 pandemic. We found that community cohesion is substantially positively associated with transportation and rehabilitation equipment rental services as well as high levels of satisfaction with community physicians’ technical expertise and community waste disposal. Community cohesion is also directly or indirectly associated with the physical and mental health of older adults. Our research suggests that enhancing community services and environmental resources is an effective strategy to increase community cohesion during major infectious disease epidemics. Improving community cohesion will also help to promote the physical and mental health of older adults.

## Abbreviations

DASC-21: 21-item Dementia Assessment Sheet for Community-Based Integrated Care System
OARS: Older American Resources and Services
OR: odds ratio

## Data Availability

The data sets generated and analyzed during this study are not publicly available to ensure the privacy of participants but are available from the corresponding author on reasonable request.
